# Pennsylvania

**Published:** 1896-04

**Authors:** 


### PENNSYLVANIA STATE VETERINARY MEDICAL ASSOCIATION.

The fourteenth annual meeting assembled in Odd Fellows' Temple, Broad and Cherry Streets, Philadelphia, March 3d and 4th. The meeting was called to order March 3d at 10.30 A.M., President Pearson in the chair. The following members were present at the various sessions: Drs. Adams, Allen, Benner, Bridge, Butterfield, Castor, Collins, Conard, Diemer, Ferley, Fitzpatrick, Felton, Foelker, Good, Goentner, Harger, Hartman, John R. Hart, Walter Hart, Hoffman, F. F., Hoskins, Houldsworth, Jobson, Keller, Keil, Knight, Kooker, Larzelere, Lintz, Lusson, Michener, Marshall, Miller, McAnulty, McClellan, Nicholson, Noack, Oyler, Pearson, G. B. Rayner, T. B. Rayner, James B. Rayner, John B. Raynor, Rechtenwald, Reinhart, Rhoads, Ridge, Ross, Salinger, Stanton, Senseman, Schreiber, Terry, Turner, Williams, Zuill. Visitors: Dr. H. P. Eves, of Delaware; Dr. W. B. Atkinson, editor and publisher of *Public Health*, Philadelphia; Dr. C. E. Magill, of New Jersey; Drs. F. C. McCurdy and J. C. McBirney, of Philadelphia; Dr. Harry B. Rayner, of Norristown, Pa.; Mr. G. H. Stokes and Mr. Teufel, of Philadelphia; and veterinary students Shaw, Land, Martien, Bertram, Mohler, Ely, Repp, Beatty, Gardiner, Jones, Lushington, Cunningham, Chesley, Weinberg, Johnson, McClure, and Kirby.

The President delivered his annual address, touching many points, more especially upon the many great changes our profession has experienced during the past year, the marvellous growth of veterinary schools, and the number of additions to our fold, the domain of the veterinarian, the value of expert veterinary advice insufficiently appreciated through lack of opportunities to draw upon the knowledge and skill of veterinarians, etc., because he has specialized to such an extent upon the diseases of the horse that his broader domain is not sufficiently recognized; he also spoke upon the growing importance of meat-, milk-, and dairy-inspection. The Chair announced that nominations and election for officers to serve for the ensuing year were in order, when the following selections were made: President, W. H. Ridge; 1st Vice-President, James B. Rayner; 2d Vice-President, J. T. McAnulty; 3d Vice-President, J. T. Ferley; Treasurer, John R. Hart; Recording Secretary, W. G. Benner; Corresponding Secretary, F. S. Allen; Board of Censors, S. J. J. Harger, W. L. Zuill, W. H. Hoskins, J. C. McNeil, T. B. Rayner.

Corresponding Secretary Allen then read the following applications for membership: Drs. George B. Jobson, Charles Falconer, and A. O. Cawley. McNeil and Timberman, of the Board of Censors, being absent, the Chair appointed Drs. James B. Rayner and W. S. Kooker to fill the vacancies. A recess of fifteen minutes was taken that the Board of Censors might examine the applications. The Board being ready to report, the chairman offered a favorable recommendation of Drs. A. O. Cawley, George B. Jobson and Charles Falconer. Dr. Jobson being present was introduced by the President. He thanked the Association with a few well-chosen remarks. A motion prevailed that each applicant favorably recommended shall be elected by acclamation, which was so conducted, and Drs. Cawley, Jobson, and Falconer were elected members.

The Corresponding Secretary then made his report. It was moved and seconded that the report be accepted and filed; carried.

Dr. W. H. Hoskins announced the death of Dr. Custer, eulogizing the deceased, and moved that a committee be appointed to draft suitable resolutions; carried. Drs. Hoskins (Chairman), T. B. Rayner, and Otto Noack were appointed.

Dr. John R. Hart, Treasurer, was then called upon for a report as to the condition of the treasury, to which he responded as follows: Total receipts, $308.53; expenses, $195.00; balance in treasury, $113.53. Moved and seconded that a committee be appointed to audit the accounts. The Chair appointed Drs. Zuill, Harger, and Ferley.

Under the head of unfinished business, Dr. Hoskins reported that the Committee on Resolutions had forwarded a letter to Mr. Woodring, Northampton County, but that it had not been acknowledged. Report of Committee on Resolution received and committee discharged.

Dr. Hoskins also reported the continued illness of Dr. S. E. Weber; that he was in a pitiable condition, suffering from great nervous prostration; that the contribution at last fall's meeting amounted to $26, which he gratefully acknowledged; that he was being cared for by a kind farmer, a former client.

Dr. Harger reported a membership certificate of Dr. Charles Williams in his possession which was placed on the table for disposal. He could not recollect the cause of its being in his possession. The Chair then appointed Drs. Zuill, Harger, and Kooker to investigate the matter, with power to act.

Under new business, Dr. Ridge offered the following: *Resolved*, That Article VII., Section 1, shall be altered to read: "These By-laws may be altered or amended by two-thirds of the members present at the annual meeting, provided the said alterations or amendments have been proposed in writing at a previous meeting by two members, and any article or section of these By-laws may be suspended during a single meeting by the unanimous consent of the members present, except the suspension be to vote on the alteration or amendment of the By-laws." W. H. Ridge, W. L. Zuill. Discussion followed as to the best method to be empioyed to collect initiation fees and dues. Dr. Hoskins moved that the Secretary comply with the report and recommendations, etc., of the Treasurer. After considerable discussion, in which several participated, the motion was carried.

At this hour, 1.15 P.M., a motion for adjournment for luncheon was entertained. After which all present repaired to higher realms through the medium of the elevator, where luncheon had been spread by the Philadelphia members. [To be concluded in the May number.]

*Officers and Committees for 1896–97.* President, W. H. Ridge, Trevose, Bucks Co.; First Vice-President, James B. Rayner, West Chester; Second Vice-President, James T. McAnulty, 1447 South Eighth St., Philadelphia; Third Vice-President, J. T. Ferley, 1431 South Sixth St., Philadelphia; Treasurer, John R. Hart, 2577 Amber St., Philadelphia; Recording Secratary, W. G. Benner, Doylestown, Bucks Co.; Corresponding Secretary, F. S. Allen, 800 North Seventeenth St., Philadelphia.

Board of Censors: Chairman, W. H. Hoskins, 3452 Ludlow St., Philadelphia; S. J. J. Harger, Thirty-sixth and Pine Sts., Philadelphia; Wm. L.

Zuill, 857 North Broad St., Philadelphia; J. C. McNeil, 26 Fourth St., Pittsburg; Thomas B. Rayner, Chestnut Hill, Philadelphia.

Committee on Legislation: Chairman, John R. Hart, 2577 Amber St., Philadelphia; Leonard Pearson, 2024 Pine St., Philadelphia; J. C. Foelker, 119 South Seventh St., Allentown; Jacob Helmer, Scranton; Charles T. Goentner, Bryn Mawr; S. J. J. Harger, Thirty-sixth and Pine Sts., Philadelphia; Thomas B. Rayner, Chestnut Hill, Philadelphia.

Committee on Intelligence and Education: Chairman, Wm. L. Zuill, 857 North Broad St., Philadelphia; F. S. Allen, 800 North Seventeenth St., Philadelphia; J. C. Michener, Colmar; M. J. Collins, Myerstown; Robert Gladfelter, 703 North Eleventh St., Philadelphia; J. W. Sallade, Pottsville; Charles Williams, 1321 Thompson St., Philadelphia.

Committee on Sanitary Science and Police: Chairman, Leonard Pearson, 2024 Pine St., Philadelphia; M. E. Conard, West Grove; W. H. Knight, Kennett Square; P. H. Minster, Langhorne; C. R. Good, Lock Haven; Charles Schauffler, 915 Dauphin St., Philadelphia; Otto Noack, Sixth and Cherry Sts., Reading.

County Secretaries: Allegheny, McNeil; Berks, Noack; Bucks, Turner; Chester, Conard; Crawford, McLean; Dauphin, Oyler; Delaware, Lintz; Erie, Irons; Fayette, Magee; Franklin, Jobson; Jefferson, Hoffman; Lackawanna, Helmer; Lancaster, Bachman; Lebanon, Collins; Lehigh, Foelker; Luzerne, Timberman; Lycoming, Kellar; Montgomery, Lusson; Northampton, Radley; Northumberland, Cawley; Philadelphia, Felton; Schuylkill, Sallade; Susquehanna, Butterfield; Washington, McKenna; Wyoming, Stanton.

### ECHOES OF THE KEYSTONE CONVENTION.

The election of Dr. W. H. Ridge as President is a well-deserved honor, and will keep the work of the organization well in touch and progress with sister associations over the country.

The reëlection of Secretaries Allen and Benners will keep well-tried men in office, and with the experience already attained through arduous labors will make the work easier for them and more effective for the Association.

Professor Zuill promises at an early date to give to the Association his well-considered views on the use of tuberculin and other agents for diagnostic purposes, as well as the dangers that may follow their use, from his point of view.

The home of Ex-Secretary Gladfelter has been gladdened by the arrival of a son, which fully accounts for the doctor's tardiness at the opening hours of the session.

The local Committee of Arrangements was somewhat alarmed at the number in attendance on the second day's session, when seventy-three presented themselves for luncheon, and only fifty-five had been provided for, but they proved equal to the emergency and saw that none went away hungry.

The introduction of a half-day's entertainment around the operating arena of the Veterinary Department of the University of Pennsylvania proved a strong drawing card.

Dr. F. F. Hoffman in exhibiting a spaying instrument fashioned after his own ideas, and constructed through his own mechanical handiwork, demonstrated its worth and practicability by quickly and efficiently spaying a mare before those in attendance.

Ethyl chloride, a new local anæsthetic, was exhibited by member Hoskins, and a brief report of its use in three cases was given, in two of which with great satisfaction, and no retarding of the healing process subsequently ; in the third case, the removal of a fibro-bony tumor from the jaw of a horse, the anæsthesia was not as complete as one would desire.

The ever-faithful Rayner family was represented each session by the five brothers, who form a very useful and strong contingent of the State Association at every call made upon them for aid and support in every direction.

Philadelphia veterinarians turned out in great strength and found a large number of those outside the city to greet them.

What occurred to Messrs. Ridge and Benners during the night of the first day has not yet been explained; suffice it to say that their friends were alarmed the next morning when one of the morning dailies had their photographs labelled wrong. The members were somewhat relieved when, at roll-call, they both responded and appeared well physically.

It added much to the pleasure of the Convention to greet the ever-faithful members, Drs. Foelker, Rectenwald, and Reinhart. Few meetings are ever held without noting their presence.

It is to be hoped that the rigid examination of the baby member, George B. Jobson, was no factor in his reported illness before arriving home.

Ex-President Pearson was somewhat embarrassed in demonstrating the value and promptness of barium chloride as a purgative when his students submitted to him a mare that had been through the experience so often that she had become injection-proof to its actions, and very knowingly looked at him out of one corner of its eye while he impatiently waited for the profound results aforesaid described. His second subject proved more satisfactory in results, though he took the precaution to inject him outside, and someone was skeptical enough to say that a wheelbarrow had been used to convey the large amount just outside the door of the amphitheatre.

The operation of cunean tenotomy for spavin-lameness by Dr. Zuill was much admired and the dexterity of its performance commended and appreciated. Another time it is hoped that better provision may be made by which a larger proportion of those present may see the *modus operandi* of this plan of surgical treatment.

One of the principal personages announced for a place on the programme was startled to find, on the eve of leaving home for the Convention, that his dimensions physically had so increased, owing, we suppose, to the lack of exercise incident to a dull winter's professional work, that his convention clothes would not button at several important points. It is to be hoped that the spring work and summer heat will remedy this misfortune in time for our fall meeting.

Median neurotomy, by Dr. Harger, was closely watched by all those who have had such varied experience in results from this operation. With some it now holds a higher and stronger place than ever, because of less frequent unfortunate complications in the aggregate than were generally looked for and realized.

New Jersey sent but one of her representatives, Dr. W. B. E. Miller, an always strong association man. This State was close enough that distance could play but a small excuse for so few being present.

All in all, it was good to be there. None regret the efforts put forth to attend. Barring illness or death, they will all be there next year. The absent ones lost much, and from one letter received, printed on another page of this issue, we are sure that he needs to be present at every meeting.

